# How Does Self-Sacrificial Leadership Foster Knowledge Sharing Behavior in Employees? Moral Ownership, Felt Obligation and Supervisor-Subordinate *Guanxi*

**DOI:** 10.3389/fpsyg.2022.910707

**Published:** 2022-07-08

**Authors:** Xiaofeng Su, Xiaoli Jiang, Guihua Xie, Meijiao Huang, Anxin Xu

**Affiliations:** ^1^College of Business Administration, Fujian Business University, Fuzhou, China; ^2^College of Marxism, Minjiang University, Fuzhou, China; ^3^College of Economics and Management, Fujian Agriculture and Forestry University, Fuzhou, China

**Keywords:** self-sacrificial leadership, knowledge sharing, moral ownership, felt obligation, supervisor-subordinate *guanxi*

## Abstract

Recent trends in the academic literature indicate growing interest in leadership for fostering employees' knowledge sharing. In this research, we integrate social cognitive theory and social exchange theory to explain how and when self-sacrificial leaders promote employee knowledge sharing. It is found that self-sacrificial leaders influences employees' knowledge sharing by nurturing employee moral ownership and a sense of obligation to the organization. We also found the moderating effect of supervisor-subordinate *guanxi* on the self-sacrificial leadership—employees' knowledge sharing link. We discuss the implications of these findings for understanding and promoting self-sacrificial leaders and employees' knowledge sharing behaviors in the workplace.

## Introduction

In today's economy, knowledge has been considered as unique strategic source for organizational innovation, value transmission and the achievement of sustainable competitive advantages (Grant, 1996; Cabrera and Cabrera, 2005). In organizations, most managers would encourage employees to sharing knowledge. However, things usually go contrary to one's wishes. Many employees withhold their knowledge or play “dumb.” Such phenomenon is called “knowledge hiding.” Knowledge management consists of five processes, i.e., knowledge acquisition, knowledge coding and storage, knowledge sharing, knowledge creation and knowledge application (Hendriks, 1999; Sedighi et al., 2016). Emerging research has found that knowledge sharing is one of the biggest challenges. The knowledge required for organizational innovation and performance is mastered by individual employees, while the organization does not own employees' “knowledge/intellectual assets.” Therefore, it has become a great challenge for leaders or managers to force employees to share knowledge with other members in the organization.

Many scholars have examined relevant factors that promote or hinder knowledge sharing from organizational level and individual level (Li et al., 2014; Xu et al., 2020). Yet, our understanding of how leaders foster employees' knowledge sharing remains quite limited. Leadership is a core feature of organizations—it is very difficult to think about organizations without thinking about who leads or manages them, and about how well they are led and managed (Hogg and van Knippenberg, 2003). Arguably, effective leadership is good at inspiring employees to adopt values, attitudes, and goals, and to behave in ways that serve the organization as a collective, and that define membership of the organization (Burns, 1978; Bass, 1985). Thus, emerging studies have begun to discuss how different leadership styles influence employees' knowledge sharing (Wu, 2021). However, what kind of leadership is effective to stimulate employees' knowledge sharing is a question worth exploring.

Among the various types of leadership styles, self-sacrificial leadership shares similar connotations with employees' knowledge sharing, both of which places organizational interests before personal interests. According to social learning theory, “people are most likely to follow their role models if the role models' behavior produces valued outcomes, rather than unrewarding or punishing effects” (Bandura, 1977). This suggests that the impact of self-sacrificing leaders' impact on employees' knowledge sharing involves more than simple behavioral emulation. Despite these indications, we have limited insights into through what kind of psychological processes, self-sacrificial leaders, in their capacities as role models, shape employees' knowledge sharing, and under what conditions these relationships hold. Previous study have found that leaders who sacrifice themselves for organizations' benefits may inspire employees' ethical cognition (e.g., moral ownership) and felt obligation (e.g., responsibility, duty) (Den Hartog, 2015). Hence, this paper aims to examine the relationship between sacrificial leadership and the critical dual mediating roles of moral ownership and felt obligation. What's more, in the Chinese environment, another factor contributing to the effectiveness of managing staffs cannot be ignored. That is building and maintaining good *guanxi* (i.e., interpersonal connection) between managers and employees (Law et al., 2000). Therefore, we also incorporated the variable of supervisor-subordinate *guanxi* as the situational feature into the research framework, and examined the moderating effect of this variable on the relationship between self-sacrificial leadership and knowledge sharing, so as to clarify the boundary conditions of the theoretical model and enhance the research's effectiveness.

## Hypothesis Development

### Moral Essence of Self-Sacrificial Leadership

Self-sacrifice refers to an individual's willingness to suffer losses in order to uphold his beliefs and values (Yorges et al., 1999). The self-sacrificial leader at an organization shows that in the process of realizing the goals and missions of the organization, leaders are not afraid of the loss of personal interests and actively contribute to the interests of the organization. It is regarded as a typical characteristic of excellent leaders (De Cremer and van Knippenberg, 2004). Choi and Mai-Dalton (1998, 1999) were the first to systematically carry out the research on the self-sacrificing behavior of leaders, and proposed the concept of self-sacrificial leadership, which was defined as “leaders' total/partial abandonment, and/or permanent/temporary postponement of personal interests, privileges, or welfare” in the (1) division of labor, (2) distribution of rewards, and (3) exercise of power (Choi and Mai-Dalton, 1999).

Specifically, in terms of division of labor, self-sacrificial leaders reject personal comfort and safety, and they proactively take on high-risk or arduous work tasks, and take responsibility for misfortunes, failures, accidents, or mistakes in the organization or team. In terms of distribution of rewards, in order to maintain or promote collective interests, leaders do not hesitate to give up or delay the acquisition of individual legitimate interests, such as welfare, salary or bonus. In terms of the exercise of power, self-sacrificial leaders do not use power for personal gains, and they are willing to give up or limit the privileges and enjoyment of individual positions.

Through three studies (one questionnaire and two laboratory experiments), Mulder and Nelissen (2010) found that self-sacrificial leaders demonstrate their ethics and cooperation motives and significantly enhance individual's response to social dilemmas, that is, they will prompt employees to strive beyond personal goals and contribute to collective goals and interests. In this sense, self-sacrificial leadership can be regarded as a series of moral behaviors. Emerging researches have shown that leaders play a key role in shaping employees' work behaviors, decision making, and work situations (Shao et al., 2011; Li et al., 2014). That is, employees may enact prosocial behaviors or workplace deviance because they observe and subsequently emulate the behaviors of role models in leadership positions.

### Moral Essence of Knowledge Sharing

Knowledge sharing can be considered an important moral issue. Bavik et al. (2017) also point out that it is necessary and important to employ a moral lens, in order to explore how to foster knowledge sharing. In workplaces, to participate in knowledge sharing, especially tacit knowledge sharing is to consciously follow a course of action with the knowledge that doing so might incur risk or threat to the self. Employees that engage in knowledge sharing behavior may experience the loss of competitive advantages, professional authority and personal interests (Li et al., 2014; Su et al., 2018, 2021). Generally speaking, as a non-institutional arrangement, employees who participate in knowledge sharing can't receive explicit rewards. Therefore, although this kind of behavior can stimulate new ideas or methods, there will be various costs to actually implement it. These possibilities make it potentially challenging for leaders to foster in employees.

According to previous literature, knowledge sharing is a series of behaviors in which employees selectively transfer or contribute their acquired knowledge or professional skills to other members within an organization (Ipe, 2003; Saeed et al., 2022). But, researchers have also found that individuals usually face the “social dilemma” of whether to share or hide knowledge, and in most cases, individuals instinctively tend to hide and store knowledge rather than share their own knowledge (Liu and de Frank, 2013; Lu and Tu, 2018). On the one hand, knowledge sharing requires a lot of time and energy from the sharer. And tacit knowledge is the source of individual's competitive advantage in an organization. Hence, sharing knowledge may lead to the loss of individual competitive advantages or personal authority (Su et al., 2018). At the same time, due to the low visibility and quantification of knowledge sharing, those who actively share knowledge are difficult to obtain equivalent rewards from the organization. From a rational point of view, the cost of knowledge sharing is much higher than the benefit, which goes against individuals' rationality. On the other hand, parts of knowledge, especially tacit knowledge, cannot be observed and recorded (Lu and Tu, 2018), and there is no formal penalty for employees who do not share knowledge. If employees tend to hide their knowledge instead of sharing, it may lead to negative impact on both employees' and organization' innovation, productivity and performances (Abdullah et al., 2019; Anser et al., 2021). So, knowledge sharing can also basically be considered as a moral challenge.

### Self-Sacrificial Leadership, Moral Ownership, and Knowledge Sharing

According to social learning theory (Bandura, 1977), leaders set an example for their subordinates based on their authority and role norms. Front-line supervisors, in particular, are prime models because of their proximity to and frequency of interactions with employees (MacKenzie et al., 1999). They draw the attention of their subordinates when they set work objectives, emphasize behavioral standards, monitor progress, and provide feedback (Ogunfowora et al., 2021). Thus, daily interaction provides ample opportunities for subordinates to observe and imitate the leader's behavior (positive or negative). This may be more pronounced in the context of collectivist culture. Because in collectivist culture, individuals are more sensitive to interpersonal information, and their psychology and behavior are more susceptible to the influence of other people in the group, especially those important ones (e.g., direct leaders in the organization) (Tian and Li, 2015). In workplaces, how leaders deal with the situations they face and how they treat their subordinates usually defines their attributes of leadership (Zada et al., 2022a).

In the research fields of leadership, many leadership styles are similar with self-sacrificial leadership, such as transformational leadership, charismatic leadership, ethical leadership, and servant leadership. All of these leadership styles involve more or less sacrificing connotation. However, these leadership theories are relatively broad, vague or general in their specific descriptions of self-sacrificing behavior, while self-sacrificial leadership shows more specific, detailed, systematic and in-depth acts of leaders' self-sacrifice (Zhou and Long, 2017). Self-sacrificial leaders would emphasize that the mission and goals of the organization are the most important. Moreover, they focus on collective wellbeing, and may even give up their personal interests in order to achieve collective interests (Choi and Mai-Dalton, 1999; Zada et al., 2022b). Self-sacrificial leaders may become role models of employees, and exercise referential power over them, thereby motivating them to exhibit similar behaviors that advance the collective good (Li et al., 2014). We argue that knowledge sharing falls into this category of behavior. When leaders adopt a self-sacrificial leadership style, employees are more likely to exhibit knowledge sharing behaviors that promote the improvement and enhancement of collective wellbeing. Thus, we posit the following hypothesis.

**Hypothesis 1**. Self-sacrificial leadership is positively related to employees' knowledge sharing.

Moral motivation is an important cognitive process that precedes moral action. Hannah and Avolio (2010) posit that the motivation to engage in ethical behavior begins when an individual takes responsibility for an ethical action in a given situation. That is, it is necessary to form a sense of moral ownership to initiate and motivate moral action. When leaders act as ethical role models, they actually show their subordinates how to successfully take ownership of the ethics of one's work context (Ogunfowora et al., 2021). Previous research has shown that leaders are substantially connected with knowledge sharing behaviors and have pivotal responsibilities in the success of the business by psychologically influencing workers to spread information (Wu and Lee, 2017; Saeed et al., 2022). When individuals emulate self-sacrificial leaders' behaviors, they will acquire and then internalize their moral values, standards and belief system, and thus forming a sense of psychological moral ownership. Thus, we posit the following hypothesis.

**Hypothesis 2**. Self-sacrificial leadership is positively related to employees moral ownership.

Once psychological ownership of moral values and standards becomes more strongly integrated into the sense of self, individuals will possess a strong desire to protect and maintain it (Pierce et al., 2003). Individuals with a strong sense of moral ownership will be less likely to “turn a blind eye” when tacit knowledge sharing is necessary for the development of an organization (Hannah et al., 2011). We propose that act of knowledge sharing is a behavioral response motivated by a desire to maintain internal consistency and sense of self by protecting the moral standards that one “owns.” Hence, feelings of ownership are associated with a willingness to give up personal interests and sacrifice in the service of caring for and protecting the collective benefits of the organization. Thus, we posit the following hypothesis.

**Hypothesis 3**. Moral ownership mediates the relationship between self-sacrificial leadership and knowledge sharing.

### Self-Sacrificial Leadership, Felt Obligation, and Knowledge Sharing

Felt obligation is defined as an individual's belief about whether he/she cares about the organization's wellbeing and helps to achieve the goals of organization (Eisenberger et al., 2001), which reflects and embodies the culturally universal norm of reciprocity principles in social interaction. As social agents, individuals are constantly under the influence of social norms that indicate the established or approved ways of thinking and behaving. According to Liang et al. (2012), individuals internalize the norm of reciprocity mainly through social learning, which constitutes a strong motivational drive (Liang et al., 2012). Researchers posit that self-sacrificial leaders have a strong sense of obligation to fulfill the responsibilities to their subordinates and the organization and to ensure that subordinates' welfare and goals of the organization are ultimately achieved. Leaders' self-sacrificing behaviors and the associated personal losses and risks, also indicate a leader's high commitment to group or organizational goals and missions (Van Knippenberg and Hogg, 2003). According to the social learning theory, the self-sacrificing behavior of the leader will strengthen the employee's perception and awareness of the organization's responsibility through role modeling. Thus, we posit the following hypothesis.

**Hypothesis 4**. Self-sacrificial leadership is positively related to felt obligation.

In turn, employees will consider self-sacrificial leaders' benefit as a form of psychosocial support and generate positive emotions and attitudes for the role modes. So, they will feel an obligation to reciprocate by doing things that benefit the leader and the organization (Baranik et al., 2010). Literature on the formation of duty in organizations (McAllister and Ferris, 2016) holds that individuals act intentionally to benefit the collective (e.g., organization and work group) out of a sense of obligation that originates in basic principles of social exchange and mutual interdependence. Driven by a strong sense of responsibility to the organization, employees will be more proactive to explicit behaviors that are beneficial to the organization (Eisenberger et al., 2001). Applied to the current context, we posit that self-sacrificial leadership encourages employees to engage in knowledge sharing by fostering a nuanced sense of obligation to help the organization achieve its goals despite potential loss of advantages to the self. Extensive research highlights the direct contribution of knowledge sharing to the goals and successes of the organization, which is a manner consistent with the moral principles upheld and modeled by the self-sacrificial leader. Combined with the viewpoint of social exchange theory, this study proposes that when faced with the above-mentioned behaviors of self-sacrificial leaders, employees will not only have positive evaluations and perceptions of leaders, but also attribute them to the organization the leader represents, and reduce their fear of being “exploited” by the organization, and generate a sense of obligation to reward and actively contribute to the organization by sharing tacit knowledge with their colleagues. Thus, we posit the following hypothesis.

**Hypothesis 5**. Felt obligation mediates the relationship between self-sacrificial leadership and employees' knowledge sharing.

### The Moderating Role of Supervisor-Subordinate *Guanxi*

In workplaces, supervisor-subordinate *guanxi* is defined as an informal personal relationship between superiors and subordinates through non-working interactions for instrumental purposes in fulfilling the collective objectives and interests (Law et al., 2000; He et al., 2020; Yang and Yang, 2020). Many authors have argued that *guanxi* is of particular relevance in managing Chinese staff. Compared with other countries, the overlap between work and social relations is much more pervasive in China. It is extremely hard for Chinese employees to imagine working in an organization in the absence of broad or far-reaching personal interactions with co-workers (Law et al., 2000). Superior-subordinate *guanxi* in the Chinese context is usually built through non-work factors. It is an integration of contract and identity relationships, and has clear hierarchical differences.

Social exchange theory holds that the interaction between leaders and employees can be regarded as a social exchange relationship (Blau, 1964). Specifically, the code of conduct and advice of well-connected leaders can more easily influence the behavior of employees. Frequent personal interactions with supervisors provide ample opportunity for employees to gain insight into leaders' sacrificing behaviors for the long-term interests of the organization and also helps employees understand how the organization works. Based on the principle of reciprocity, employees will show behaviors that are beneficial to the development of the team and organization to repay the trust and support of leaders (Gao and Liu, 2021). Hence, employees will be more likely to share knowledge in order to contribute to the goals of the organization. On the contrary, employees with weaker *guanxi* between superiors and subordinates have no way to deeply understand the leader's code of self-sacrificial conduct, and it is also difficult to obtain the leader's personal guidance, and may be reluctant to share tacit knowledge. Thus, we posit the following hypothesis.

**Hypothesis 6**. Supervisor-subordinate *guanxi* moderates the direct effects of self-sacrificial leadership on employees' knowledge sharing. Specifically, the direct effects are positive and stronger when supervisor-subordinate *guanxi* is better and weaker when supervisor-subordinate *guanxi* is worse.

**Figure 1:**
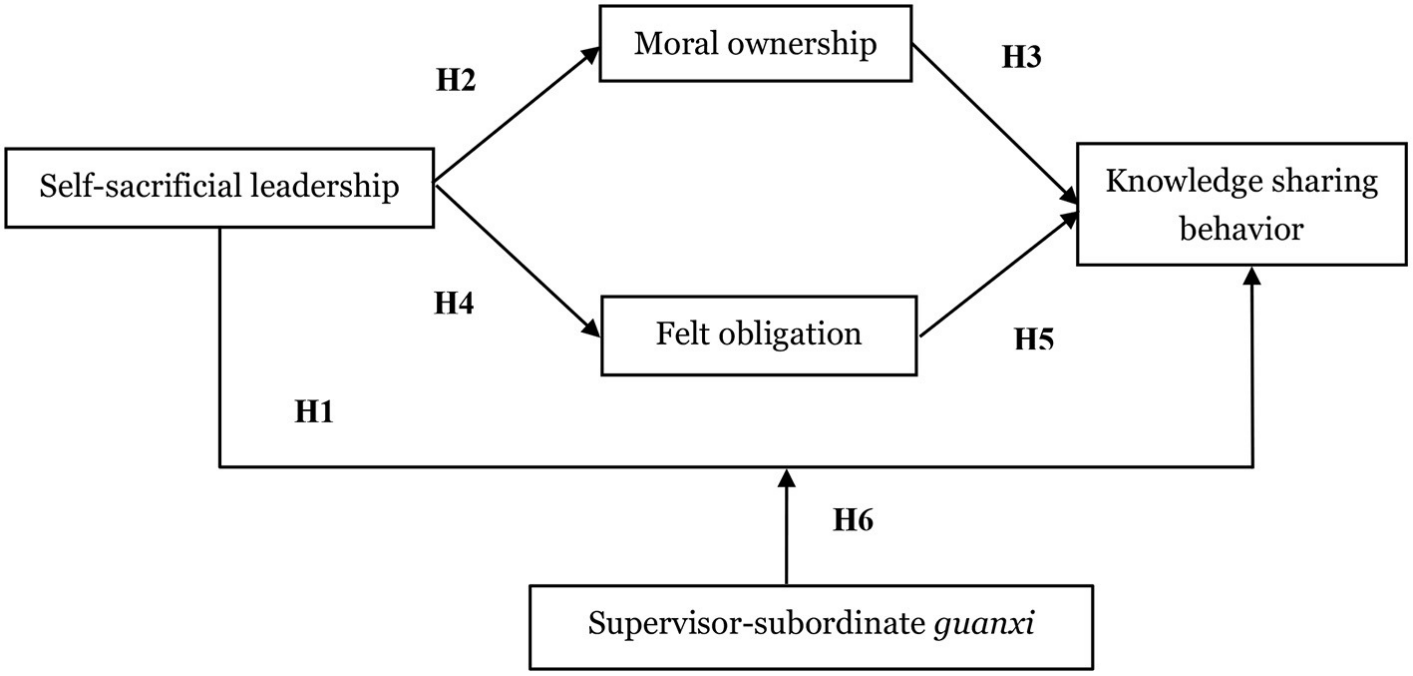
Conceptual model.

Based on the above analysis, the theoretical model in this paper is shown in Figure 1.

## Methods

### Procedure and Participants

This study collects data through questionnaire survey. All of the respondents are postgraduate students of MBA (Master of Business Administration) courses from three universities in Fujian, China. Those who are full-time employees and currently have a direct supervisor are invited to participate in the survey and those who have no direct supervisor were not excluded. To avoid the effect of common method bias, this study collected relevant data from two sources. We collected data at two different points in time to strengthen the ability to make a causal inference and to reduce the influence of common method bias. Specifically, all the participants were given a file bag with two envelopes inside, which were marked differently, saying “YOU” and “YOUR SUPERVISOR.” The questionnaires contained in the envelope marked “YOU” covering the variables of self-sacrificial leadership, moral ownership, felt obligation and demographic variables. The participants were required to complete the questionnaires during the class. And the questionnaires in the envelope marked “YOUR SUPERVISOR” were the items of employees' knowledge sharing. This envelope was brought back by the participant and given to their immediate supervisor to fill in the answers, and then taken back or emailed to us after completion.

With the help of university teachers, a total of 600 pairs of questionnaires were distributed. Five hundred and seventy-three MBA students completed the “YOU” survey and 526 were valid (87.67% valid response rate). And we received 513 questionnaires from “YOUR SUPERVISOR” survey, of which 505 were valid, with an effective recovery rate of 84.17%. By matching the employee questionnaire and the supervisor questionnaire according to the pre-marked coding, 481 sets of valid questionnaires were finally obtained, with a total valid response rate of 80.17%.

The sample is composed of relatively young respondents, 93.76% of whom are under the age of 45. More than half (52.18%) of the respondents in the sample were female. About 68.81% of respondents have worked for their current companies for 5 years or more, while 31.19% of respondents have spent <5 years for their current companies. In terms of education level, all employees graduated from vocational colleges or universities.

### Measurements

The measurement scales of the key variables in this study were all adopted from the English literature. In order to ensure the accuracy of semantic connotation and the intelligibility of language expression for all items of the scale, we organized an editing group. Two overseas students were invited to participate in the group to do “translation and back translation” of the questionnaire items. The two students are good at both Chinese and English and their research field is leadership and organizational behavior. Except for control variables, participants rated their agreement on a five-point response scale (1 = strongly disagree to 5 = strongly agree). The questionnaire items of each variable were shown in Table 1.

(1) Self-Sacrificial Leadership. We used De Cremer and van Knippenberg (2004) five-item scale. Sample items are “My supervisor is willing to make personal sacrifices in the teams interest.” Reliability estimate (i.e., Cronbach's α) is 0.941.
(2) Moral ownership. We used Hannah and Avolio's (2010) three-item moral ownership scale. Sample items are “I will not accept anyone in my group behaving unethically” and “I will assume responsibility to take action when I see an unethical act.” Cronbach's α is 0.922.
(3) Felt obligation. We adapted four items from Ogunfowora et al.'s (2021) scale. Example items included “I feel a personal obligation to do whatever I can to help my (supervisor) organization achieve its goals” and “I would feel an obligation to take time from my personal schedule to help my (supervisor) organization if it needed my help.” Cronbach's α is 0.935.
(4) Knowledge sharing. Knowledge sharing behavior of the employees was reported by their direct supervisor using Connelly et al. (2012) five-item scale. Cronbach's α is 0.928.
(5) Supervisor-subordinate *guanxi*. Employees were asked to rate their likelihood of exhibiting the behaviors/activities proposed by four items developed by Law et al. (2000). Cronbach's α is 0.940.

**Table 1:** Discriminant validity and common bias test.

**Constructs**	***X* ^2^**	**df**	**X^**2**^/df**	**GFI**	**CFI**	**NFI**	**RMSEA**
Hypothesized five-factor model (SAC; MOR; OBL; SHAR; GUAN)	729.611	189	3.860	0.811	0.955	0.940	0.077
Five-factor model (SAC + MOR; OB; SHAR; GUAN)	1256.153	193	6.509	0.784	0.911	0.897	0.107
Three-factor model (SAC + MOR + OBL; SHAR; GUAN)	1404.031	196	7.163	0.774	0.899	0.884	0.113
Six-factor model (SAC; MOR; OBL; SHAR; GUAN; CMV)	550.643	167	3.297	0.907	0.968	0.955	0.069

*SAC, self-sacrificial leadership; MOR, moral ownership; OBL, felt obligation; SHAR, knowledge sharing; GUAN, supervisor-subordinate guanxi; CMV, common method variance*.

Control variables. Referring to previous related studies (Li et al., 2014; Tian and Li, 2015), we selected common demographic variables in organizational behavior, i.e., gender, age and working tenure as control variables.

### Statistical Methods and Analytical Ideas

All statistical analyses were performed using SPSS 21.0 and Amos 24.0 in this study. First, confirmatory factor analysis was used to test the discriminant validity of the relevant scales. Secondly, SPSS 21.0 was used for descriptive statistical analysis; Finally, hierarchical regression analysis was used to examine self-sacrificial leadership, the relationship between leadership trust and employee knowledge sharing, and the moderating role of employee tradition.

## Results and Brief Discussion

### Discriminant Validity and Common Bias Test

To test the discriminant validity between variables, the goodness of fit of each competing factor model was compared by confirmatory factor analysis. The results in Table 1 show that the fitting indicators of the five-factor model (GFI = 0.811; CFI = 0.955; TLI = 0.940; RMSEA = 0.077) all meet the empirical standard and are significantly better than other factor models, which indicates that the research variables have good discriminant validity.

Referring to the research proposal of Podsakoff et al. (2003); Tang and Wen (2020), we tested the common method bias by adding a common method variance (CMV) to the hypothetical model. The results in Table 1 show that after adding CMV, the fitness effect of the model is not significantly improved compared with the five-factor model, which indicates that the common method bias would not seriously interfere with the research conclusions. Therefore, this five-factor model was used for the further assessment of the structural model in the next step.

### Reliability and Validity Tests

Before testing the research hypotheses, we assessed the reliability and validity of the measurement model. Reliability reflects the level of internal consistency of each construct in the research model, which is generally characterized and measured by Cronbach's α coefficient, CR (composition reliability) and AVE (average variance extraction). The results in Table 2 shows that both the Cronbach's α coefficients and CR of all the latent variables are >0.7, and the AVE value is >0.6 (Fornell and Larcker, 1981). All of the values have reached the threshold of reliability requirements, which indicates an acceptable result for the reliability and convergent efficiency test.

**Table 2:** Means, standard deviations, AVE and correlations among study variables.

**Construct**	**Mean**	**S.D**.	**Cronbach's α**	**CR**	**AVE**	**SAC**	**MOR**	**OBL**	**SHAR**	**GUAN**
SAC	4.413	0.517	0.941	0.944	0.773	**0.879**				
MOR	4.342	0.555	0.922	0.922	0.799	0.764^**^	**0.894**			
OBL	4.335	0.566	0.935	0.936	0.784	0.745^**^	0.768^**^	**0.885**		
SHAR	4.352	0.519	0.928	0.932	0.732	0.823^**^	0.869^**^	0.821^**^	**0.856**	
GUAN	4.335	0.547	0.940	0.941	0.801	0.742^**^	0.820^**^	0.782^**^	0.855^**^	**0.895**

*S.D., standardized deviation; CR, composition reliability; AVE, average variance extracted. SAC, self-sacrificial leadership; MOR, moral ownership; OBL, felt obligation; SHAR, knowledge sharing; GUAN, supervisor-subordinate guanxi. The bold face diagonal values are the square roots of AVE. The values in the lower triangle are the Pearson correlation coefficients between the latent variables*;

** *p < 0.01*.

Table 2 also shows the correlation coefficients of all the variables. It can be seen that the square root of the AVE value for each construct is greater than its correlation coefficient with the remaining variables, indicating that discriminant validity of the overall scale is supported.

### Descriptive Statistics and Correlation Analysis

The descriptive statistics and correlation analysis of variables are shown in Table 2. The results show that self-sacrificial leadership is significantly positively correlated with knowledge sharing (β = 0.823, *p* < 0.01), moral ownership (β = 0.764, *p* < 0.010), and felt obligation (β = 0.745, *p* < 0.01). Moral ownership (β = 0.869, *p* < 0.01) and felt obligation (β = 0.821, *p* < 0.01) are also significantly positively correlated with knowledge sharing. The results are basically consistent with the expectation of the aforementioned research hypothesis.

### Hypothetical Test

This study uses the hierarchical regression analysis method to test the aforementioned hypothesis, and the results are shown in Tables 3, 4. We follow the three steps proposed by Baron and Kenny (1986) to test to the impact of self-sacrificial leadership on employees' knowledge sharing (Hypothesis 1), moral ownership (Hypothesis 2) and felt obligation (Hypothesis 4) as well as the mediating roles of moral ownership and felt obligation (Hypothesis 3 and Hypothesis 5). First, the relationship between the independent variable (self-sacrificial leadership) and the outcome variable (knowledge sharing) was examined. Then, the effect of the independent variable (self-sacrificial leadership) on the mediating variables (moral ownership and felt obligation, respectively) were tested. Finally, by adding the mediating variable, we tested whether the impact of the independent variable (self-sacrificial leadership) on the outcome variable (knowledge sharing) changed significantly.

**Table 3:** Results of direct and mediating effect test.

	**Knowledge sharing**		**Moral ownership**	**Knowledge sharing**	**Felt obligation**	**Knowledge sharing**
**Construct**	**M1**	**M2**	**M3**	**M4**	**M5**	**M6**
Gender	−0.114^*^	−0.048	−0.011	−0.050^*^	−0.020	−0.047^*^
Age	0.088	0.021	0.046	0.030	0.014	0.050^*^
Work tenure	−0.163^**^	−0.091^**^	−0.006	−0.013	−0.019	−0.007
Salary	−0.090^*^	−0.017	−0.024	−0.009	0.060	−0.051^*^
Self-sacrificial Leadership		0.804^***^	0.762^***^	0.378^***^	0.728^***^	0.473^***^
Moral ownership				0.573^***^		
Felt obligation						0.469^***^
*R* ^2^	0.067	0.69	0.586	0.820	0.560	0.780
Adj. *R*^2^	0.059	0.687	0.582	0.817	0.556	0.778

* *p < 0.05*;

** *p < 0.01*;

*** *p < 0.001*.

**Table 4:** Results of moderating effect test.

	**Knowledge sharing**			
**Construct**	**M8**	**M9**	**M10**	**M11**
Gender	−0.108	−0.057^*^	−0.024	−0.017
Age	0.131^**^	0.056^*^	0.036	0.041^*^
Work	−0.050	−0.016	0.010	0.007
Salary	0.100^*^	−0.023	0.011	0.007
Self-sacrificial Leadership		0.814^***^	0.415^***^	0.444^***^
Supervisor-subordinate *guanxi*			0.540^***^	0.495^***^
Leadership × Supervisor-subordinate *guanxi*				0.110^***^
*R* ^2^	0.053	0.684	0.813	0.824
Adj. *R*^2^	0.045	0.680	0.810	0.821

* *p < 0.05*;

** *p < 0.01*;

*** *p < 0.001*.

#### Direct Effect Test

The developed hypotheses (shown in Figure 1) were tested by running regression analysis in SPSS21.0. Table 3 shows the results of direct effect and indirect effect. In this part, knowledge sharing is added as the dependent variable to test the control variables and self-sacrificial leadership's direct impact on knowledge sharing. First, we examined the impact of the three control variables, i.e., gender, age and working tenure on knowledge sharing. Model 1 shows that employees' gender (β = −0.114, *p* < 0.05), working tenure (β = −0.163, *p* < 0.01) and salary (β = −0.090, *p* < 0. 05) have significant negatively impacts on knowledge sharing. Based on M1, we included self-sacrificial leadership as the independent variable in Model 2. It is found that, self-sacrificial leadership (β = 0.804, *p* < 0.001) has a positive impact on knowledge sharing. Hence, hypothesis 1 is supported.

#### Mediating Effect Test

We further tested the mediating effect of moral ownership and felt obligation according to the procedures introduced by Wen et al. (2004), which has been used widely by scholars. The results have also been shown in Table 3. As we have tested the direct effect of self-sacrificial leadership and employees' knowledge sharing, we further examined the relationship between self-sacrificial leadership and the two mediating variables. As shown in M3 and M5, the independent variable self-sacrificial leadership is significantly related to the moral ownership (β = 0.762, *p* < 0.001) and felt obligation (β = 0.728, *p* < 0.001). Thus, Hypothesis 2 and 4 are both supported.

Based on M3 and M4, we add the two mediating variables, i.e., moral ownership (β = 0.573, *p* < 0.001) and felt obligation (β = 0.469, *p* < 0.001), both of which are significantly correlated with knowledge sharing. And, it is also found that the correlations between self-sacrificial leadership and knowledge sharing in M4 and M6 are still significant although their coefficients decreased. It indicates that both moral ownership and felt obligation have significant positive mediating effects between self-sacrificial leadership and employees' knowledge sharing. Hence, hypothesis 3 and 5 are supported.

#### Moderating Effect Test

Regarding the moderating effect of supervisor-subordinate *guanxi* on the relationship between self-sacrificial leadership and knowledge sharing, this study adopts the procedures suggested by Baron and Kenny (1986). Before we conducted moderate analysis, we *z*-scored the data of independent variable (self-sacrificial leadership) and the moderating variable (supervisor-subordinate *guanxi*). After that, we calculated the interactive variable (Leadership × Supervisor-subordinate *guanxi*). The results of moderating effect are shown in Table 4. M9 and M10 shows that both self-sacrificial leadership and supervisor-subordinate *guanxi* have significant impacts on knowledge sharing. And M11 shows that the interactive variable has a significantly positive impact on knowledge sharing, which indicated that supervisor-subordinate *guanxi* plays a significant effect between self-sacrificial leadership and employees' knowledge sharing. Hence, Hypothesis 6 is supported.

## General Discussion

The present research was motivated by our limited understanding of how leader self-sacrificial leadership influences employee's knowledge sharing. We empirically tested two alternate explanations derived from social cognitive and social exchange theories. Across the empirical study, we show that self-sacrificial leaders influence employee knowledge sharing by fostering in employees intrinsic moral ownership and an extrinsic sense of obligation (to the supervisor and organization). We also consistently find that supervisor-subordinate *guanxi* strengthened the direct effect of self-sacrificial leadership on employees' knowledge sharing.

### Theoretical Implications

These findings have a number of theoretical implications. First, our research highlights the complementary roles that social learning and social exchange processes play in understanding how self-sacrificial leaders promote employees' knowledge sharing. In essence, self-sacrificial leadership is an embodiment of a leader's high level of moral character (Choi and Mai-Dalton, 1998). And Chinese social culture has always attached great importance to the moral quality of leaders (Ling et al., 1987). The results show that self-sacrificial leadership still has important value and plays an positive role in today's Chinese companies. The observation that self-sacrificial leaders encourage employees to share knowledge instead of hiding aligns with Bandura's (1977) point that intrinsic motivation is critical to understanding how leaders' role model influence employees' behavior. In addition, employees do not simply emulate behaviors from their leaders. Rather, they also learn from to develop personal moral ownership for some prosocial actions. Once moral ownership is acquired, it is difficult for employees to turn a blind eye when their supervisor or organizations need them to share knowledge for collective goals.

Second, our study shows that self-sacrificial leaders also foster employees' knowledge sharing by inculcating a strong sense of obligation to help their supervisors or the organization achieve collective performance or interests. To date, most scholars have largely overlooked the possibility that leaders may promote employees' knowledge sharing through social exchange-based processes. Our study extends the theoretical lens through which scholars can understand this phenomenon. Our findings show that in addition to cultivating intrinsic moral ownership for knowledge sharing, employees also enact such behavior out of a sense of moral duty or obligation for collective benefits.

Third, we show that although self-sacrificial leadership influence employees' knowledge sharing, the strength of these relationships depends, in part, on supervisor-subordinate *guanxi*. When subordinates has good *guanxi* with their leaders, they tend to have a better understanding of their leaders' self-sacrificial behaviors. They will be more willing to share tacit knowledge to promote the development of their organization.

### Practical Implications

Our findings have practical implications for organizations. First, organizations should care about the personal character of the leaders because employees tend to learn from their supervisors as their role models. Self-sacrificial leaders' role modeling fosters in employees the motivation and obligation to share knowledge for organizational benefits rather than hide it just for personal interests. Given that supervisors are salient ambassadors of the organization, organizations must exercise sound judgment when identifying or selecting individuals who are likely to possess the characteristics necessary to be effective models (Ogunfowora et al., 2021). Therefore, organizations can establish effective procedures and systems to screen and promote managers with a self-sacrificial leadership style, and develop this style of the managers through leadership training activities.

Second, our research also suggest that leaders can use two distinct strategies for promoting employees' knowledge sharing, i.e., by nurturing an intrinsic moral ownership and/or by cultivating their sense of obligation to help the organization to achieve collective goals. Training and development exercises should be implemented to teach leaders how to boost the ownership and sense of duty in their employees. For managers, they should often review their own leadership behaviors or methods, and strive to take the lead beyond self-interest and do their best for the collective interests and wellbeing, so as to motivate employees to actively contribute their strength and wisdom to the operation and development of the organization, and ultimately create the organization's competitive advantage and success.

Third, in order to stimulate employees knowledge sharing, organizations should promote the establishment of good relations between leaders and subordinates by providing leaders with relevant training, and improve their awareness and ability to establish good relations with subordinates. At the same time, organizations can also organize various activities to increase the opportunities for leaders to communicate with their subordinates and enhance their friendship. For leaders, they should care about the needs of employees in daily work, actively strengthen effective communication with employees, provide opportunities for them to undertake challenging tasks, and encourage and inspire employees to do some more creative work.

## Limitations

Although our research has drawn some meaningful conclusions, there are still some limitation. First, this study focuses on the impact of self-sacrificial leadership on employees' individual behavior. In fact, the effect of self-sacrificial leadership can be further extended to the team level. Future research can examine the impact of self-sacrificial leadership on teams or departments. Second, we conducted the research in China, in which the cultural influence, e.g., on collectivism and the spirit of self-sacrificing, may be more manifest. Future research can compare the findings in different countries and regions. Finally, further research should consider other possible moderating variable.

## Data Availability Statement

The raw data supporting the conclusions of this article will be made available by the authors, without undue reservation.

## Ethics Statement

Ethical review and approval was not required for the study on human participants in accordance with the local legislation and institutional requirements. The participants provided their written informed consent to participate in this study.

## Author Contributions

XS, MH, and AX contributed to the conception and design of the study. GX organized the database. XS performed the statistical analysis. XS and XJ wrote the first draft of the manuscript. MH and GX wrote sections of the manuscript. All authors contributed to manuscript revision, read, and approved the submitted version.

## Funding

The paper was supported by Fujian Social Science Foundation for Young Scholars (FJ2021C093), Fujian Innovation Strategy Research Project (2021R0092), National Education Science 13th Five-Year Plan 2017 Youth Ministry of Education The Impact of the Matching Degree of College Tutors and Postgraduates on the Cultivation of Innovative Talents and Countermeasures (EIA170466).

## Conflict of Interest

The authors declare that the research was conducted in the absence of any commercial or financial relationships that could be construed as a potential conflict of interest.

## Publisher's Note

All claims expressed in this article are solely those of the authors and do not necessarily represent those of their affiliated organizations, or those of the publisher, the editors and the reviewers. Any product that may be evaluated in this article, or claim that may be made by its manufacturer, is not guaranteed or endorsed by the publisher.

## Appendix

**1. Self-sacrificial leadership**

(1) My supervisor is willing to make personal sacrifices in the teams interest.
(2) My supervisor is willing to stand up for the team members interest, even when it is at the expense of his/her own interest.
(3) My supervisor is willing to risk his/her position, if he/she believes the goals of the team can be reached that way.
(4) My supervisor is always among the first to sacrifice free time, privileges, or comfort if that is important for the teams' mission.
(5) I can always count on my supervisor to help me in times of trouble, even if it is at costs to him/her.

**2. Moral ownership**

I will not accept anyone in my group behaving unethically.
I will assume responsibility to take action when I see an unethical act.
I will not tolerate anyone in my group who violates safety standards.

**3. Felt obligation**

(1) I feel a personal obligation to do whatever I can to help my (supervisor) organization achieve its goals ethically (achieve safety goals).
(2) I owe it to my (supervisor) organization to give 100% of my energy to attaining its goals in an ethical manner (its safety related goals while I am at work).
(3) I owe it to my (supervisor) organization to do what I can to ensure that my work is completed in an ethical manner (ensure that our workplace is safe).
(4) I would feel guilty if I did not meet my (supervisor's) organization's ethical standards (safety standards)

**4. Knowledge sharing**

(1) This coworker looks into my requests to make sure his/her answers were accurate.
(2) This coworker explains everything very thoroughly.
(3) This coworker answers all my questions immediately.
(4) This coworker tells me exactly what I need to know.
(5) This coworker goes out of his/her way to ensure that he/she understands my requests before responding.

**5. Supervisor-subordinate**'**s** ***guanxi***

(1) During holidays or after office hours, I would call my supervisor or visit him/her.
(2) I always actively share with my supervisor about my thoughts, problems, needs and feelings.
(3) I care about and have a good understanding of my supervisor's family and work conditions.
(4) When there are conflicting opinions, I will definitely stand on my supervisor's side.
